# Cardiopulmonary Events of the Elderly (≥75 Years) during Clazosentan Therapy after Subarachnoid Hemorrhage: A Retrospective Study from a Tertiary Stroke Center in Japan

**DOI:** 10.3390/medicina60010185

**Published:** 2024-01-21

**Authors:** Tatsushi Mutoh, Hiroaki Aono, Wataru Seto, Takehiro Kimoto, Ryota Tochinai, Junta Moroi, Tatsuya Ishikawa

**Affiliations:** 1 Department of Surgical Neurology, Research Institute for Brain and Blood Vessels, Akita Cerebrospinal and Cardiovascular Center, Akita 010-0874, Japan; 2 Department of Aging Research and Geriatric Medicine, Institute of Development, Aging and Cancer, Tohoku University, Sendai 980-8575, Japan; 3 Department of Pharmaceutics, Akita Cerebrospinal and Cardiovascular Center, Akita 010-0874, Japan; 4 Department of Veterinary Pathophysiology and Animal Health, Graduate School of Agricultural and Life Sciences, The University of Tokyo, Tokyo 113-8654, Japan

**Keywords:** clazosentan, cerebral vasospasm, elderly, subarachnoid hemorrhage, systemic events

## Abstract

Clazosentan has been shown to prevent vasospasm and reduce mortality in patients after aneurysmal subarachnoid hemorrhage (SAH) and has been approved for clinical use in Japan; however, its systemic events in the elderly (aged ≥ 75 years) have not been well-documented. Here, we report serious/intolerable cardiopulmonary complications requiring discontinuation of drug therapy in elderly SAH patients. In this single-center case series study, medical records of consecutive SAH patients treated postoperatively with clazosentan (10 mg/h) between June 2022 and May 2023 were reviewed retrospectively. Thirty-three patients received clazosentan therapy, of whom six were elderly with a mean age of 80.3 ± 5.2 (range 75–89) years. Among them, despite no obvious medical history of systemic abnormalities, clazosentan was discontinued in three (50%) patients due to pleural effusion and hypoxemia with or without hypotension at 5 ± 3 days after therapy initiation, which was higher than the incidence for younger patients (15%). The elderly patients had significantly lower urine output (1935 ± 265 vs. 1123 ± 371 mL/day, *p* = 0.03) and greater weight gain (2.1 ± 1.1 vs. 4.2 ± 1.9 kg from baseline, *p* = 0.04) than patients who completed the therapy. One 89-year-old female developed congestive heart failure and hydrostatic pulmonary edema associated with increased intravascular and lung volumes even after therapy was discontinued, while the remaining two cases recovered within 2 days after drug cessation. These results suggest that elderly patients are more vulnerable to fluid retention and have a higher risk of cardiopulmonary complications during clazosentan therapy than younger patients. Careful monitoring of urine volume and weight gain and caution regarding age- and therapy-related hemodynamic insufficiencies are required.

## 1. Introduction

Aneurysmal subarachnoid hemorrhage (SAH) accounts for approximately 3% of all strokes worldwide [1]. The incidence of SAH varies by countries from 4 to 28 per 100,000 person-years, and Japan has been reported to have the highest incidence in the world [2]. The risk of death and permanent disability increases if the patient develops cerebral vasospasm, which can lead to delayed cerebral ischemia (DCI) within 2 weeks of ictus, after surviving the initial bleeding and related early brain injury [3], thus requiring prompt treatment to avoid brain infarction and related poor outcomes [4,5]. In addition, SAH can also cause central-nervous-system-mediated secondary systemic complications such as Takotsubo cardiomyopathy and neurogenic pulmonary edema early after SAH [6,7,8]. Previous studies have suggested that hypoxemia due to acute post-SAH pulmonary edema may lead to a global decrease in cerebral perfusion and ischemia, thereby complicating fluid therapy for treating cerebral vasospasm and DCI thereafter [9].

Currently, nimodipine, a dihydropyridine calcium channel blocker, remains the only drug recommended universally in this patient population [10]. As a novel pharmacological prevention strategy of post-SAH DCI, a fast-acting selective endothelin receptor antagonist clazosentan has been investigated extensively [11,12,13,14]. Based on the results from phase 3 trials to reduce cerebral vasospasm incidence and related morbidity at 6 weeks [15], clazosentan obtained initial approval for clinical use in Japan in 2022. Although prior studies have described the efficacy and safety profile of clazosentan (i.e., well-tolerated but increased rate of lung complications, anemia, and hypotension) [16], only limited data are available in elderly patients (≥75 years), in which no statistical significant differences were detected compared to younger patients [17]. However, older patients are prone to having severe neurological and systemic dysfunctions attributable to brain vulnerability, underlying comorbidities, and a high risk of cardiopulmonary complications after SAH [18]. Therefore, it is conceivable that we need to pay more attention to fluid therapy using clazosentan, particularly for elderly SAH patients.

In this case series, we describe the systemic events that could lead to therapy discontinuation in elderly patients receiving clazosentan as a first-line anti-vasospasm therapy. When compiling this work, we particularly focused on serious/intolerable cardiopulmonary complications and analyzed changes in related routine measurements. Furthermore, we followed cases where, in patients with persistent hemodynamic and/or respiratory insufficiencies, intensive systemic management with the use of advanced hemodynamic parameters (i.e., cardiac output, intravascular volume, extravascular lung water, and pulmonary permeability index) from transpulmonary thermodilution monitoring was indicated [19].

The medical records of 33 consecutive patients with aneurysmal SAH admitted to the emergency room of the Akita Cerebrospinal and Cardiovascular Center between June 2022 and May 2023 were considered for retrospective analysis. The diagnosis of aneurysmal SAH was confirmed via non-contrast computer tomography (CT) followed by exploring the location of the aneurysm using contrast-enhanced CT angiography. Information relating to comorbidities was sourced from previous medical statements of the patient and from doctors’ reports after admission, where information regarding their health status could be obtained.

As per the manufacturer’s instructions, all patients received clazosentan as a continuous intravenous infusion at 10 mg/h, starting within 48 h postoperatively after SAH (onset; designated as day 0) for up to 14 days, with a minimum of 10 days. Vasodilatory and/or antiplatelet agents such as nimodipine (not approved in Japan), fasudil hydrochloride, or cilostazol were not used. Levetiracetam (1000 mg/d) was administered to prevent postoperative epilepsy. Intracranial pressure was controlled within the normal range using cisternal, ventricular, or cerebrospinal drainage, as necessary. Hyponatremia (serum sodium level of <130 mEq/L) or hypokalemia (serum potassium level of <3.0 mEq/L) was corrected by adding each electrode solution (10 mL of 10%NaCl or K^+^ 10 mEq) to the main fluid bag. If hyponatremia persisted, fludrocortisone (0.1 mg/d) was given. Blood transfusion was performed only when the hematocrit level was <30%. If the maximal systolic blood pressure was >180 mm Hg, a calcium antagonist was administered. For adverse cardiopulmonary events nonresponsive to standard medical care within 48 h, clazosentan was discontinued and switched further to rescue therapy, including dobutamine-induced hyperdynamic and/or endovascular therapy [19,20].

The primary outcome of this study was discontinuation of clazosentan therapy. Demographic, clinical, and radiological data were compared between patients with completed and those with discontinued clazosentan therapy. Treatment-necessitating serious adverse events were documented. Therapy was discontinued upon adverse event onset (e.g., hypotension and respiratory failure) or due to a lack of efficacy (disease progression requiring rescue therapy). Along with routine hemodynamic monitoring and laboratory/neurological exams, body weight, urine volume, and fluid balance were measured daily. In addition to arterial blood gas exams, chest X-ray, head CT, and echocardiography were performed when cardiorespiratory insufficiency was suspected. Secondary outcomes included occurrence of DCI, 90-day functional outcomes, and discontinuation of clazosentan therapy. Evaluation for radiological vasospasm and DCI (newly developed infarction) was performed via CT angiography, magnetic resonance angiography, and/or diffusion-weighted magnetic resonance imaging on days 5–7 and 14–16 after SAH. Transpulmonary thermodilution (PiCCO; Getinge, Gothenburg, Sweden) was used for advanced hemodynamic monitoring. The transpulmonary thermodilution system requires injections of a bolus of cold saline in the superior vena cava territory either via a central venous catheter or a peripherally inserted central catheter line. At the tip of a femoral arterial catheter, a thermistor senses the decrease in blood temperature to calculate measurements of cardiac output, global end-diastolic volume (as an index of preload), extravascular lung water (equivalent to a measure of pulmonary edema), and pulmonary vascular permeability index [7,21,22]. The full details regarding set-up and measurements for SAH patients were provided in our previous study [23]. Functional outcomes were assessed based on modified Rankin Scale (mRS) scores (favorable: 0–2; poor 3–6).

Data were checked for normal distribution with the Shapiro–Wilk test. Continuous variables were compared using the unpaired *t*-test or Mann–Whitney *U* test for normally distributed or skewed data, respectively. Categorical variables are presented as frequencies and percentages and were compared using Fisher’s exact test. The variables showing statistically significant differences between completed and discontinued groups were entered into multivariate logistic regression analysis to detect parameters associated with the discontinuation of clazosentan in the univariate analyses. All analyses were performed using Prism (version 9: GraphPad Software, La Jolla, CA, USA). Data were presented as mean ± standard deviation (range) or number (%) unless stated otherwise. *p*-values < 0.05 were considered statistically significant.

## 2. Case Report

### 2.1. Clinical Data Description

**Figure 1:**
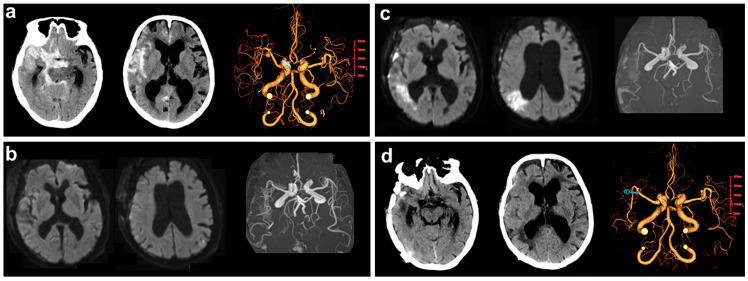
Imaging findings. (**a**) Head CT scans on admission in an 89-year-old patient with SAH showing ruptured right middle cerebral artery aneurysm and intra-sylvian hematoma. Although postoperative MR diffusion-weighted images and angiography performed on day 7 were unremarkable (**b**), new infarction and distal vasospasm were detected in the posterior trunk territory of the right MCA (**c**), suggestive of cerebral vasospasm and related DCI. (**d**) Follow-up CT scan on day 21.

**Figure 2:**
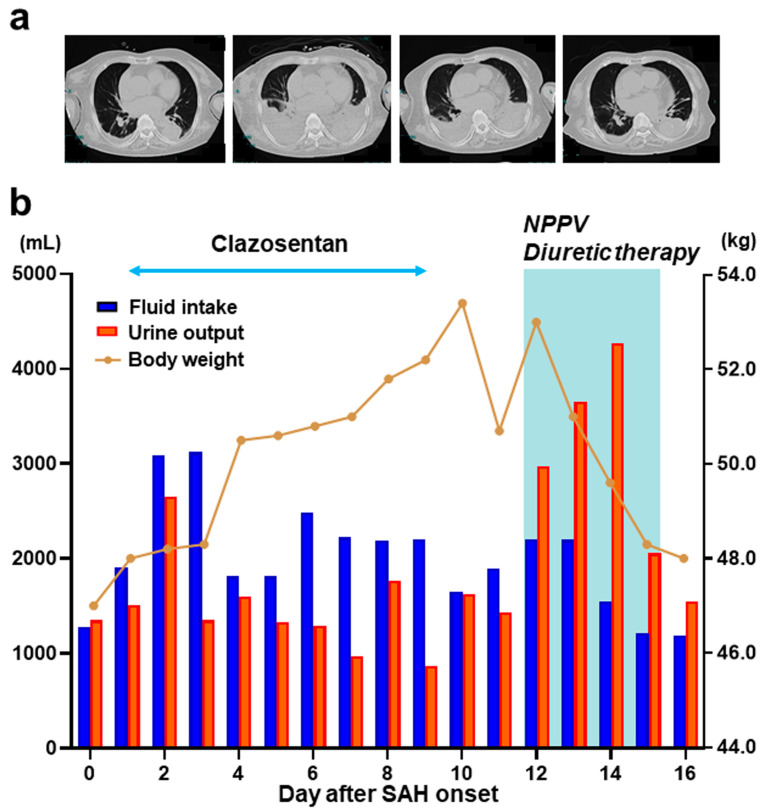
Computed tomography findings and fluid retention parameters in an 89-year-old woman with SAH. (**a**) Serial chest computed tomography scans obtained on admission and days 9, 14, and 21 after SAH onset; (**b**) temporal changes in daily fluid intake, urine output, and body weight during clazosentan therapy (double arrow) and intensive care including NPPV and diuretic therapies assisted by the transpulmonary thermodilution device after the discontinuation (color bar). NPPV, noninvasive positive-pressure ventilation.

**Figure 3:**
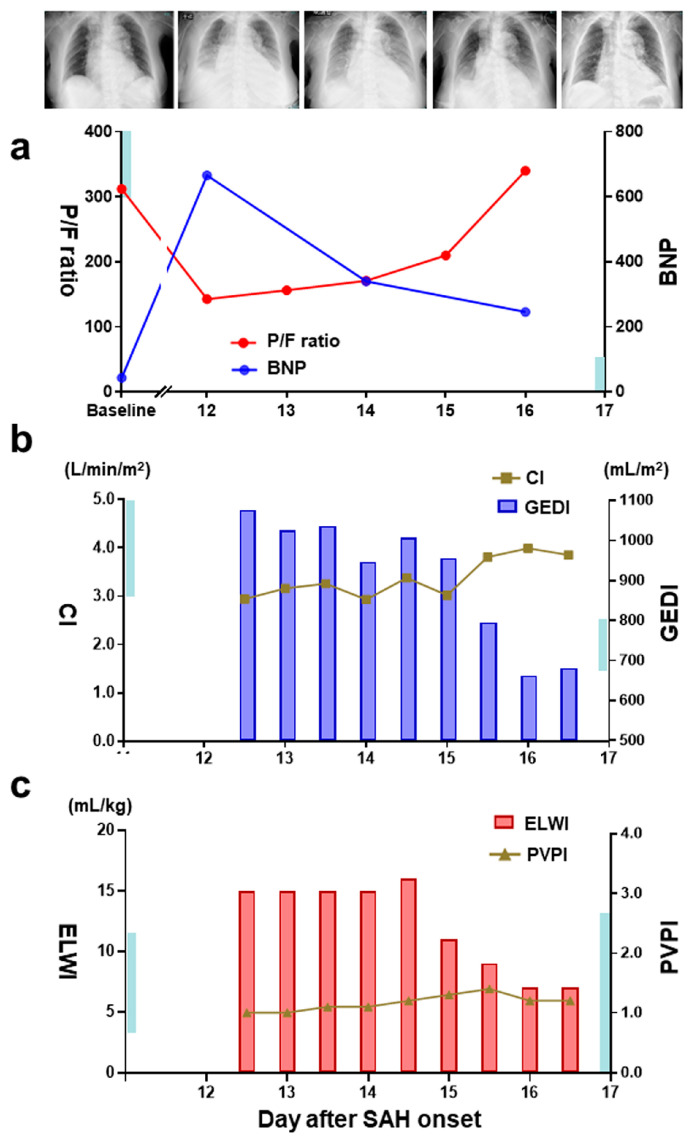
Chest X-ray findings and hemodynamic parameters in the same patient. (**a**) Chest X-ray images obtained on admission and days 12, 14, 16, and 21 after SAH onset; (**b**) temporal changes in the ratio between PaO2 and the fraction of inspired oxygen (P/F ratio) and serum B-type natriuretic peptide (BNP) levels, (**c**) transpulmonary thermodilution-derived cardiac index (CI) and global end-diastolic volume index (GEDI), and (**c**) extravascular lung water index (ELWI) and pulmonary vascular permeability index (PVPI). The small bar in each Y-axis indicates the normal reference limits.

We provide a summary of clinical data (Table 1) and systemic events related to the drug discontinuation (Table 2) of each elderly patient, a summary of general clinical data between completed and discontinued clazosentan therapy in Table 3, and detailed descriptions of case no. 6 (Figure 1, Figure 2 and Figure 3) complicated with irreversible respiratory and hemodynamic deterioration even after the cessation of clazosentan therapy. For reference, a summary of clinical data for all SAH patients (*n* = 33) is presented in Supplementary Table S1.

### 2.2. Case Series Summary

Of the 33 patients who received clazosentan, 6 were aged ≥ 75 years. Among them, three (50%) had a history of vascular comorbidities but were independent in their daily life activities (Table 1). Their radiological and echocardiographic findings on admission were unremarkable. All six patients underwent successful aneurysm obliteration under general anesthesia within 12 h of SAH ictus, and there were no perioperative systemic complications.

Clazosentan was discontinued in three (50%) patients due to hypoxemia and pleural effusion with or without hypotension at 5 ± 3 (range, 3–9) days after therapy initiation. Among these, one patient (case no. 6) subsequently developed congestive heart failure due to increased intravascular volume, further complicated with acute pulmonary edema and aspiration pneumonia even after therapy discontinuation, whereas in the remaining two patients (Table 2), all symptoms resolved within 48 h.

Compared with those who completed therapy, patients who discontinued therapy had significantly lower urine output and greater weight gain. Renal function, as assessed with the estimated glomerular filtration rate trend, was maintained. Although angiographic vasospasm in distal blood vessels was observed in most patients (83%), clazosentan therapy discontinuation did not affect DCI incidence or 90-day functional outcomes (Table 3).

### 2.3. Illustrative Case

An 89-year-old woman (case no. 6) with a medical history of hypertension and mild dementia was admitted to the emergency room due to a sudden loss of consciousness. On admission, the patient was comatose and did not follow any commands (Glasgow Coma Scale score, 6). Head CT revealed severe subarachnoid hemorrhage combined with right intrasylvian hematoma and acute hydrocephalus (Figure 1a). After surgical clipping of a ruptured right middle cerebral artery aneurysm and hematoma evacuation, she gradually became alert without motor deficits. Clazosentan therapy was started on postoperative day 1. Although she had no remarkable hemodynamic insufficiency or neurological/radiological finding suggestive of DCI (Figure 1b), urine volume gradually declined after day 4, with increasing body weight and fluid balance (Figure 2b).

Despite fluid titration and intermittent furosemide injections to maintain normovolemia, we had to discontinue clazosentan infusion due to intolerable hypoxemia and rapidly progressive pleural effusion detected on day 9 (Figure 2a). However, hypoxemia persisted in the next 2 days even after drug cessation. Echocardiography revealed a dilated inferior vena cava in the absence of any cardiac involvement. In conjunction with high B-type natriuretic peptide levels (Figure 3a), we assumed the development of congestive heart failure secondary to increased intravascular volume, which may further deteriorate the pleural effusion contributing to persistent hypoxemia.

Therefore, we decided to assess precise volumetric and hemodynamic parameters for further treatment. The data derived from transpulmonary thermodilution revealed high levels of EVLW and GEDI without PVPI elevation accompanied by low CI, suggestive of hydrostatic edema (Figure 3b,c). Noninvasive positive-pressure ventilation was applied to improve the hypoxemia. Carperitide followed by tolvaptan used for volume control resulted in reduced pleural effusion and normalized intravascular/extravascular lung fluids after day 15. The patient showed cerebral vasospasm and DCI (Figure 1c) with mild left-side hemiparesis and cognitive decline and was transferred 3 months later to a long-term care hospital with an mRS score of 3 (Figure 1d).

## 3. Discussion

The main findings of this case series are that elderly patients are more vulnerable to fluid retention and have higher risks of cardiopulmonary complications that discontinue post-SAH clazosentan therapy than younger patients, and that in elderly patients, both reduced urine volume and weight gain may become predictors of additional systemic deteriorations. According to data currently available from subgroup analysis of a retrospective outcome study in Japanese patients, the occurrence of systemic effects during clazosentan therapy is similar between elderly (≥75 years) and younger (<75 years) patients and well-tolerated among both age groups [17]. However, no practical solutions have been recommended in the elderly population. In our institutional cohort data (33 consecutive SAH patients aged 37–89 years) within the current study period, clazosentan was discontinued in 21% of total patients because of hypoxemia (*n* = 7) associated with pleural effusion, pulmonary edema, and/or congestive heart failure; dysphagia associated with head and facial swelling (*n* = 2); and persistent hypotension (*n* = 3) refractory to medical treatment. In this series, the rate of drug discontinuation was extremely high in the elderly patients (50% (elderly) vs. 15% (younger)). The results were somewhat different from previous data. Fortunately, these extracranial complications improved within 48 h after discontinuation of therapy, except in one patient complicated with congestive heart failure and pneumonia requiring intensive care to resolve fluid retention and hypoxemia.

It is likely that the underlying mechanism of fluid retention may be associated with endothelin (ET)_A_ selective receptor antagonism. Clazosentan is a selective endothelin ET_A_ receptor antagonist for intravenous use with a 1000-times-higher binding affinity for the ET_A_ receptor than that for the ET_B_ receptor [16]. Although there is no supportive pharmacological evidence regarding the adverse reaction of clazosentan to cause fluid retention in SAH, other clinical data suggest that ET_A_ selective antagonists, such as ambrisentan and sitaxentan [24,25], pose a greater risk of fluid overload in pulmonary arterial hypertension than dual antagonists [26]. In a rodent pharmacological study, ET_B_ receptor overstimulation triggered by antagonizing ET_A_ receptors was a key mechanism behind fluid retention and vascular leakage [27]. These findings may help explain the high fluid retention and edema rates seen with clazosentan, particularly in conditions associated with elevated arginine vasopressin levels, such as congestive heart failure, older age, or renal dysfunction, which are frequently observed in patients with SAH, leading to reduced water excretion and increased vascular leakage. 

Compared to conventional therapy using intravenous infusions of fasudil hydrochloride, ozagrel sodium, nicardipine, and oral cilostazol during the vasospasm risk period at least until day 14 after SAH onset, clazosentan therapy increased the median weight gain and subjective fluid retention (as represented by facial edema, pleural effusion, and pulmonary edema) in elderly patients [17]. However, no specific parameters, predictors, or treatment strategies have been demonstrated regarding drug discontinuation. In our elderly consecutive cases, urine output, including daily urine volume and day-to-day variance, and weight gain were significantly different between patients with completed and discontinued clazosentan therapy. Thus, acutely reduced urine output could be an initial warning sign of fluid redistribution and related hypoxemia. Our results suggest that all potential risks must be managed by balancing potentially conflicting goals following SAH. Although clazosentan-related volumetric changes seem to be transient and reversible, therapeutic strategies should not be affected by other systemic causes hindering adequate volume retention and/or oxygenation, such as congestive heart failure and pneumonia. Recent studies have reported the importance of goal-directed therapy, guided by extended hemodynamic monitoring in patients with post-SAH extracranial complications (e.g., Takotsubo cardiomyopathy and neurogenic pulmonary edema) [6,7]. The current study supports the utility of cardiac and volumetric parameters derived from bedside transpulmonary thermodilution for distinguishing between hypovolemia and fluid retention or hydrostatic and permeability edema, and for guiding appropriate hemodynamic goals in patients with persistent hypoxemia for more than 48 h after discontinuation of clazosentan. It is interesting to note that clazosentan induced fluid retention by increasing preload volume and subsequent hydrostatic pulmonary edema without elevating lung permeability. This may explain, in part, a reversible mechanism of fluid retention and body weight gain early after discontinuation and/or by reducing intravenous fluid infusion rates [17]. On the other hand, in cases further complicated with cardiac dysfunction, as represented by case 6, washout function for both intra- and extravascular fluids may have been disrupted and thus led to the requirement for intensive diuretic (hypovolemia) therapy with respiratory support, in turn, leading to the development of post-SAH DCI.

The limitations of this study include the small sample size, retrospective nature, and its design. Because of the single-arm design, no comparison with the standard protocol or a placebo group has been demonstrated. In addition, appropriate strategies associated with clazosentan discontinuation (e.g., whether we should restart the therapy after resolution of the symptoms and its timing) should be examined in a larger population. Regarding the rescue therapy after discontinuation, the timing of the application of intensive fluid therapy using transpulmonary thermodilution and the interpretation of the derived values and treatment protocol require further confirmation. Nevertheless, given the higher rate of therapy discontinuation among older patients, our findings suggest that older patients are more vulnerable than younger adults to clazosentan-related systemic events associated with fluid retention and cardiopulmonary function deterioration.

## 4. Conclusions

To our knowledge, this is the first “real-world” data from a tertiary care center in Japan documenting adverse cardiopulmonary events among the elderly aged ≥ 75 years that could discontinue clazosentan anti-vasospasm therapy after SAH. We propose that careful monitoring of urine volume and weight gain and caution regarding age- and therapy-related hemodynamic insufficiencies are required.

## Figures and Tables

**Table 1:** Baseline characteristics of each elderly patient between completed and discontinued post-SAH clazosentan therapy.

No.	Age (y)/Sex	Medical History	WFNS Grade	Aneurysm Location	Treatment	Vasospasm	DCI	90-Day mRS
Completed								
1	75/Female	N/A	3	Left MCA	Clip	+	−	1
2	83/Male	Hypertension Diabetes mellitus	1	Left MCA	Clip	+	−	1
3	76/Female	N/A	5	Acom	Clip	−	−	4
Discontinued								
4	81/Female	N/A	3	Left MCA	Clip	+	−	2
5	78/Female	Hyperlipidemia	2	Right ICPC	Coil	+	−	1
6	89/Female	Hypertension Mild dementia	5	Right MCA	Clip	+	+	4

Cerebral vasospasm was assessed via MR angiography on days 7 and 14 after SAH and/or on the day when related clinical symptoms were suspected. DCI was determined using diffusion-weighted MR images on day 14 and with a CT scan taken around day 21. Acom, anterior communicating artery; DCI, delayed cerebral ischemia; ICPC, internal carotid-posterior communicating artery; mRS, modified Rankin scale; N/A, not applicable; MCA, middle cerebral artery; SAH, subarachnoid hemorrhage; WFNS, World Federation of Neurosurgical Societies.

**Table 2:** Cardiopulmonary complications of each elderly patient between completed and discontinued post-SAH clazosentan therapy.

No.	Age (y)/Sex	Weight Gain (kg)	Hypotension	Hypoxemia	Pleural Effusion	Congestive Heart Failure	Pulmonary Edema
Completed							
1	75/Female	+1.7	−	−	−	−	−
2	83/Male	+1.3	−	−	−	−	−
3	76/Female	+2.9	−	−	−	−	−
Discontinued							
4	81/Female	+2.9	+	+	+	+	−
5	78/Female	+3.2	+	+	+	−	−
6	89/Female	+6.4	−	+	+	+	+

Pleural effusion and pulmonary edema was diagnosed via chest X-ray and/or CT scan. Congestive heart failure was defined using echocardiography. Hypoxemia was defined with a peripheral oxygen saturation < 90% or partial arterial oxygen pressure < 60 Torr. SAH, subarachnoid hemorrhage.

**Table 3:** General clinical data for post-SAH clazosentan therapy between completed and discontinued groups among 6 elderly patients.

	Total (*n* = 6)	Completed (*n* = 3)	Discontinued (*n* = 3)	*p*
Age (y)	80.3 (5.2)	78.0 (4.4)	82.7 (5.7)	0.33
Female	5 (83)	2 (67)	3 (100)	0.50
Body weight (kg)	48.9 (3.8)	51.1 (4.6)	46.7 (0.8)	0.13
WFNS grade	3.2 (1.6)	3.0 (2.0)	3.3 (1.5)	0.85
Ejection fraction on admission (%)	66.2 (9.5)	62.3 (12.1)	71.1 (4.3)	0.33
Duration of drug therapy	9.5 (5.0)	13.7 (0.6)	5.3 (3.2)	0.02
Minimal urine volume				
24 h volume (mL/d)	1462 (537)	1935 (265)	1123 (371)	**0.04**
Volume per hour (mL/kg/h)	1.22 (0.43)	0.96 (0.37)	1.61 (0.19)	**0.02**
Day-to-day variance (mL/kg/h)	−0.64 (0.35)	−0.50 (0.37)	−0.87 (0.16)	**0.02**
Fluid balance (mL/d) at minimal urine volume	1634 (785)	1426 (1010)	1638 (832)	0.57
eGFR at minimal urine volume	70.5 (12.6)	72.0 (17.3)	68.3 (8.1)	0.57
Weight gain (kg)	3.1 (2)	2.1 (1.6)	4.2 (1.9)	**0.02**
Vasospasm	5 (83)	2 (67)	3 (100)	0.50
DCI	3 (50)	1 (33)	2 (67)	0.20
Favorable outcome (mRS 0–2) at 90 days	4 (67)	2 (67)	2 (67)	0.80

Data are shown as mean (standard deviation) or number (percentage). Statistically significant results at *p* < 0.05 are shown in bold. DCI, delayed cerebral ischemia; eGFR, estimated glomerular filtration rate; mRS, modified Rankin scale; SAH, subarachnoid hemorrhage; WFNS, World Federation of Neurosurgical Societies.

## Data Availability

The datasets generated and analyzed during the present study are available from the corresponding author upon reasonable request.

## Supplementary Materials

The following supporting information can be downloaded at: https://www.mdpi.com/article/10.3390/medicina60010185/s1, Table S1: Baseline characteristics and univariate analysis for clazosentan therapy between completed and discontinued groups among 33 consecutive SAH patients.
