# Supplementary material for: Cardiopulmonary Events of the Elderly (≥75 Years) during Clazosentan Therapy after Subarachnoid Hemorrhage: A Retrospective Study from a Tertiary Stroke Center in Japan
Source: Medicina (Kaunas). 2024 Jan 21;60(1):185. doi: 10.3390/medicina60010185 (PMC10819954; doi:10.3390/medicina60010185)
Supplement: Supplementary file 1 [file medicina-60-00185-s001.zip › Ethics approval and consent form.pdf]

秋田県立循環器・脳脊髄センター 研究所

Research Institute for Brain and Blood Vessels-AKITA

Akita Cerebrospinal and
Cardiovascular Center
6-10 Senshu-Kubota-machi
Akita 010-0874, JAPAN

TEL：+ 81-18-833-0115
FAX：+ 81-18-833-2104

Dr. Tatsushi Mutoh
Director, Second Department of Surgical Neurology
Research Institute for Brain and Blood Vessels
Akita Cerebrospinal and Cardiovascular Center, Akita, Japan

July 14, 2022

Declaration of Ethical Approval in relation to the research project "Retrospective Analysis For Systemic Events Related to Clazosentan Therapy in Elderly (≥75 Years) Patients After Subarachnoid Hemorrhage"

Letter of Declaration

This retrospective study was approved by the Ethical Committee of the Research Institute for Brain and Blood Vessels, Akita Cerebrospinal and Cardiovascular Center (**Ref No. 23-14**: Factors for discontinuation of clazosentan therapy after aneurysmal subarachnoid hemorrhage: A single-center, observational study including very elderly patients). This research involves a single injection in our arm within the range of routine medical treatment (i.e., public health insurance controlled by the Japan Health Insurance Association). Therefore, comprehensive consent has been completed by each client, patient, or legal guardian on admission.

2023年7月14日
秋田県立循環器・脳脊髄センター　病院長
石川 達哉

Tatsuya Ishikawa
Chair of the Board
Research Institute for Brain and Blood Vessels
Akita Cerebrospinal and Cardiovascular Center, Akita, Japan

倫理審査委員会の名称	■秋田県立循環器・脳脊髄センター倫理委員会<br>□その他（　　　　　　　　　　　　　　　　　　）
添付書類	■倫理審査委員会に提出した資料一式<br>■倫理審査結果通知書<br>□その他（　　　　　　　　　　　　　　　　　　）

秋田県立循環器・脳脊髄センター
収受
-5. 7. 14
C・2 A・17・5
第559号

研究等実施許可通知書

研究責任者

（所属）第二脳神経外科診療部

（職・氏名）部長　武藤　達士　　様

令和５年７月１４日に申請のありました研究等の実施について、以下のとおり通知します。

☑許可

□不許可【理由：＿＿＿＿＿＿＿＿＿＿＿＿＿＿＿＿＿＿＿＿＿＿】

□その他【内容：＿＿＿＿＿＿＿＿＿＿＿＿＿＿＿＿＿＿＿＿＿＿】

2023年7月14日

秋田県立循環器・脳脊髄センター　病院長

石川　達哉　（印）

秋田県立循環器・脳脊髄センター 研究所

Research Institute for Brain and Blood Vessels-AKITA

Akita Cerebrospinal and
Cardiovascular Center
6-10 Senshu-Kubota-machi
Akita 010-0874, JAPAN

TEL：＋81-18-833-0115
FAX：＋81-18-833-2104

包括同意について

～ 診療を受けられる患者さんへ 包括同意についてのお願い ～

Regarding the comprehensive consent

～ Request to patients who will receive medical treatment Regarding comprehensive consent ～

１．はじめに

秋田県立循環器・脳脊髄センター病院（以下「本院」という。）では，「基本理念，目標，職業倫理 方針」に則した診療を実施しております。 また，本院は診療，教育，研究を遂行する大学病院としての使命を有しています。こ のため，本院に来院する傷病者，妊婦，新生児等すべての人（以下「患者さん」という。）の人権を尊重した先進的医療を行うとともに，次世代を担う医療人（学生，研修医，看護師等）の育成も行っております。 さらに，臨床研究の場である秋田県立循環器・脳脊髄センター・研究所は，その特徴を最大限に活かして，新たな診療 法を開発する社会的使命を有しており，医学の進歩を目指して臨床研究，医学研究も行 っております。このような診療，教育，研究を円滑に進めるため，本院では，患者さんに以下の方法 を用いて「説明と同意」の確認をさせていただいております。 第一に，書面（又は口頭）で説明し，書面（又は口頭）で同意をいただく方法（個別 同意)。第二に，一定の基準の元にそのような手続きを経ないで実施する方法（包括同 意）に分けて対応しています。 ここでは，各項目に分けて包括同意に関し説明させていただきますので，本院の診療，教育，研究の役割にご理解とご協力を賜りますようお願いいたします。 また，この包括同意に関しては，ご本人からのお申し出により，診療等に支障のない 範囲内で不同意等の意思表示が可能です。なお，包括同意への意思表示の内容は，本院 で受ける診療に一切影響を与えるものではありません。

Introduction

Akita Cerebrospinal and Cardiovascular Center (hereinafter referred to as “this hospital”) provides medical care in accordance with the “basic principles, goals, and professional ethics policy.” In addition, this hospital has a mission as a university hospital that carries out medical care, education, and research. For this reason, we provide advanced medical care that respects the human rights of all individuals (hereinafter referred to as “patients”) such as the sick and injured patients, pregnant women, and newborns who visit our hospital; besides, we continue to train medical personnel (students, residents, nurses, etc.) who will lead the next

generation. Furthermore, as a public prefectural research institute, being locations for clinical research, have a social mission to make the best use of this feature and develop new medical treatment methods, and we also conduct clinical and medical research with the aim of advancing medical sciences. In order to smoothly advance such medical treatment, education, and research, we request patients to confirm "explanation and consent" using the following methods. First, a method of explanation in writing (or oral explanation) and obtaining a written (or oral) consent (individual consent). Secondly, corresponding through (comprehensive consent) that is implemented based on certain standards without going through such procedures. Here, we will explain the comprehensive consent for each item separately, so we request your 2 understanding and cooperation in the roles of medical care, education, and research at this hospital. In addition, regarding this comprehensive consent, and upon the request of the individual himself / herself, it is possible to express your intention such as disagreement, etc., within the range that does not interfere with medical treatment. The content of the manifestation of intention regarding comprehensive consent does not affect the medical treatment received at this hospital.

２．個人情報保護方針について

本院に来院する患者さんは，診療をお受けになると身体状況，病状，治療計画等の診 療情報からカルテ等の診療記録が作成されます。 この診療記録には貴重な個人情報が多く含まれております。本院では，その取扱いに つきましては，個人個人の人格の尊重を基本的な理念として持ち，取扱うことが最重要 のことであると考えており，患者さんの権利利益の保護を最優先に考え個人情報の取扱 いの適正化を図るよう努力しております。本院は,「診療，教育，研究を遂行する地域機関病院としての使命を有し，また患者さんの人権を尊重した先進的医療を行うとともに，次代を担う国際的な医療人を育成する。」と の理念のもと，本院における診療記録に含まれる貴重な個人情報を，医療機関として，また，教育研究機関として利用させていただきたいと思いますので，改めてご理解とご 協力を賜りますようお願い申し上げます。 最後に本院では，個人情報の重要性を認識し，個人情報に関する法律等を遵守することにより，個人情報保護に万全を尽くしてまいります。

Regarding privacy policy

Patients who visit our hospital will have medical records created about their medical information such as physical condition, medical condition, treatment plan, etc. while they receive medical treatment. This medical record contains many valuable personal information. We believe that respecting the personality of each individual is the basic principle and the most important issue when handling such medical records. We give top priority to the protection of the rights and interests of our patients, and we strive to optimize the handling of personal information. This hospital "has a mission as a public teaching hospital that carries out medical care, education, and research. Besides, this hospital provides advanced medical care that respects the human rights of patients, along with training international medical personnel who will lead the next generation." Based on this philosophy, we wish to use the valuable personal information contained in the medical records at our hospital as a medical

institution and as an educational and research institution. We appreciate your understanding and cooperation. Finally, at this hospital, we will do our utmost to protect personal information by recognizing the importance and complying with the law.

3. 個人情報について

Regarding personal information

本院では取得した患者さんの貴重な個人情報を含む記録を，医療機関としてだけでなく教育研究機関として所定の目的に利用させていただきますので，ご理解とご協力を賜 りますようお願いいたします。 The records including valuable personal information of patients will not be used only as a medical institution but also for the designated purpose as an educational and research institution. We request your understanding and cooperation

（1）患者さんの個人情報は，各種法令に基づいた諸規定を守ったうえで別記の目的に利用されます。 Patient's personal information will be used for the purposes described separately after observing various regulations based on various laws.

（2）上記利用目的以外に患者さんの個人情報を利用する場合は，書面により患者さんの同意をいただくことといたします。 If the patient's personal information is to be used for purposes other than the above, the patient's written consent will be obtained.

（3）患者さんに関する外部からの問い合わせ等には，次のように対応します。

・電話等による問い合わせの場合，原則としてお答えいたしません。

・入院患者さんは，お申し出いただくことで，面会を希望しないことができます。

We will respond to inquiries related to patients from outside this hospital as follows. ・ In principle, we will not answer inquiries by telephone. ・ Inpatients can request refusal of hospital visits.

（4）患者さんの同意がなければ，原則として，患者さん以外の方から情報の収集は行いません。 As a general rule, we will not collect information from anyone other than the patient without the consent of the patient.

（5）患者さんは，ご自身の個人情報に関して次の権利があります。

・患者さんは，所定の手続きのうえ，自己の個人情報の開示を請求することができま す。また，本院において以前より行っているカルテ，フィルム等の開示手続きによっても開示の請求が可能です。 なお，開示には本学所定の複写料が必要になります。

・患者さんは，開示を受けた自己の個人情報の内容について，所定の手続きのうえ， 訂正を請求することができます。

・患者さんは，自己の個人情報が不適切な取扱いをされていると思われる場合は，所 定の手続きのうえ，自己の個人情報の利用の停止，消去，提供の停止を請求するこ とができます。

・患者さんは，上記権利の決定等に関して不服がある場合は，本院に対して異議申し立てをすることができます。

Patients have the following rights regarding their personal information:

・ Patients can request disclosure of their personal information after following the designated procedures. They can also request disclosure by following the procedures for disclosing

medical records, films, etc., that have been carried out at this hospital. Note that a copy fee designated by the university is required for disclosure.

・ Patients can request corrections to the contents of their personal information that has been disclosed, following the designated procedures.

・ If the patients think that his / her personal information is being handled improperly, he / she can request the suspension of use, deletion, or suspension of provision of his / her personal information according to the designated procedures.

・ Patients can file an objection against the hospital if they are dissatisfied with the determination of the above rights.

（6）防犯カメラについて

本院では，病院の管理上，カメラや録音装置を設置しております。その記録は，病 院の管理上使用するものであり，外部に公表することを目的としたものではありません。ただし，裁判所，警察署から協力依頼があった場合は，提供することがありま す。

Regarding security cameras

At this hospital, cameras and recording devices are installed for hospital supervision. Such records are used for hospital supervision and is not intended for disclosure outside this hospital. However, if there is a request for cooperation from either the Court or police department, we may need to provide them.

（7）診療中の画像について

本院では，患者さんの治療に必要な場合は，病巣等の評価及び治療方針等を決定するため，診療中の様子をカメラ等で撮影，録音し必要な情報を得ております。これの情報は，教育研究機関として，医師等の育成等に利用することがあります。

Regarding images that are taken during medical treatment

At this hospital, when it is necessary for a patient treatment, in order to evaluate the lesion etc., and to decide the treatment policy etc., the condition during medical treatment is photographed and recorded with a camera or similar device, and necessary information is obtained. As an educational and research institution, this information may be used in the training of doctors, etc.

別記：本院における患者さんの個人情報の利用目的

Separate note: Purpose of the usage of patient's personal information at this hospital

1）本院での利用 Usage at this hospital

①患者さんがお受けになる医療サービス Medical services that patients receive ②医療保険事務 Medical insurance office work ③患者さんに関係する管理運営業務（入退院等の病棟管理，会計，経理，医療事故 等の報告，医療サービスの向上） Management and operation work related to patients (ward management such as hospitalization and hospital discharge, accounting, payment, reporting of medical accidents, improvement of medical services) ④医療サービスや業務の維持，改善のための基礎資料 As basic materials for maintaining and

improving medical services and operations

２）本院での利用 Usage at this hospital and university （以下①から③の利用に際しては，可能な限り匿名化するよう努力します。） (When using information as mentioned in ① to ③ below, we will do our best to anonymize the information as much as possible.) ①医療系教育（学生の臨床実習，医療従事者の研修） Medical education (clinical training for students, training for medical professionals) ②症例に基づく研究（研究活動を実施する際に，実施に関する法令や倫理指針，関係 団体等のガイドライン等が定められている場合は，それに沿って誠実に遂行いたしま す。） Case-based research (when conducting research activities, if there are established laws and ethical guidelines regarding implementation, guidelines of related organizations, etc. we will carry out such regulations sincerely.) ③外部監査機関への情報提供又は閲覧（法令等に基づく監査，検査，委員会等） Providing information or giving browsing rights to external auditing organizations (audits, inspections, committees, etc. based on laws and regulations)

３）他の事業者への情報提供 Providing information to other businesses ①他の病院，診療所，助産所，薬局，訪問看護ステーション，介護サービス事業者 等との医療サービス等に関しての連携 Cooperation with other hospitals, clinics, midwifery centers, pharmacies, home-visit nursing stations, nursing-care service providers, etc. regarding medical services. ②他の医療機関等からの医療サービス等に関しての照会への回答 Answers to inquiries regarding medical services, etc. from other medical institutions, etc. ③患者さんの診療等にあたり外部の医師等の意見，助言を求める場合 When seeking the opinions and advice of outside doctors when treating patients ④検体検査業務の委託，その他の業務委託 Outsourcing of sample testing business, and other business outsourcing ⑤患者さんの家族への病状説明 Explanation of the medical condition to the patient's family ⑥医療保険事務（保険事務の委託，審査支払機関へのレセプトの提出） Medical insurance affairs (outsourcing of insurance affairs, submission of receipts to examination and payment agencies) ⑦本院からの審査支払機関又は保険者への照会 Inquiries from this hospital to the examination and payment agencies or the insurer ⑧審査支払機関又は保険者からの照会への回答 Response to inquiries from examination and payment agencies or insurers ⑨関係法令等に基づく行政機関及び司法機関等への提出等 Submission to administrative and judicial organs based on relevant laws and regulations, etc. ⑩関係法令等に基づき事業者等からの委託を受けて健康診断を行った場合における事業者等へのその結果通知 Notification of the result to the business operator, etc. when a health examination is conducted on behalf of other business operators, etc. based on relevant laws and regulations. ⑪医師賠償責任保険等に係る医療に関する専門の団体や保険会社等への提供又は届出等 Provisions or notifications to medical organizations and insurance companies related to medical liability insurance, etc. ⑫臨床研修医の研修評価，医学生の臨床教育評価に係る個人情報を含まない経験症例等記録の大学病院医療情報ネットワークへの提出 Submission of records of experienced cases, etc., that do not include personal information, related to training

evaluation of clinical residents and clinical education evaluation of medical students to the medical information network of university hospitals.

３．検査，診療行為について

本院では，書面で正式な説明同意をいただく診療項目と，口頭及び掲示で説明と同意 確認をさせていただく診療項目に分けて，対応させていただいております。

About medical examination and treatment practice At this hospital, we divide the explanations and consent forms into medical care items for which formal explanation and consent are obtained in writing, and medical care items for which explanation and consent are confirmed verbally and on the bulletin board.

以下の検査，診療行為の中には，医師が立ち会う必要がないものが多く，患者さんへの心身へのご負担も少ないものです。診療を円滑に進めるために，これらの診療項目に ついては，説明と同意確認を口頭又は掲示で対応させていただいております。

Many of the following tests and medical procedures do not require the presence of a doctor, and the burden on the patient's mind and body is minimal. In order to facilitate medical examination and treatment, we will provide explanations and confirmation of consent verbally or by posting these medical treatment items on the bulletin board.

（１）【一般項目】 問診，視診，身体診察，体温測定，身長測定，体重測定，血圧測定，リハビリテー ション，栄養指導，食事の決定，カメラ等による患部撮影等

[General items] Medical interview, inspection, physical examination, body temperature measurement, height measurement, weight measurement, blood pressure measurement, rehabilitation, nutritional guidance, dietary decision, photography of diseased body parts by endoscopy and similar devices.

（２）【検査，モニター】 血液学検査（血液等の採取），尿・糞便等検査，微生物学的検査（痰・唾液等の採 取），検体の病理・細胞診検査，生理機能検査（心電図検査，脈波検査，呼吸機能検 査，脳波検査，超音波検査，筋電図検査等），Ｘ線一般撮影検査，Ｘ線透視撮影検査， 造影剤を用いないＣＴ，ＭＲＩ，ＲＩ（アイソトープ）検査，心理検査，経皮酸素飽 和度測定検査，動脈圧測定検査，モニター（ＢＩＳモニター（脳波を元に麻酔薬の効 果を推定するモニター），筋弛緩モニター等），皮内反応検査（パッチテスト，皮内テ スト，スクラッチテスト，ツベルクリン反応，最小紅斑量測定等），免疫学的検査（ウイルス検査等）

[Inspection, monitoring] Hematology test (collection of blood, etc.), examination of urine / stool, etc. microbiological test (collection of phlegm, saliva, etc.), pathological / cytological samples examination, physiological function examination (electrocardiogram "ECG", pulse wave examination, respiratory function tests, brain wave examination, ultrasonic examination "echo", electromyogram examination, etc.), X-ray general radiography examination, X-ray fluoroscopy examination, CT, MRI, RI (isotope) examination without contrast agent, psychological examination, percutaneous oxygen saturation degree measurement, arterial pressure measurement, monitor (BIS monitor (monitor that estimates the effect of anesthetics based on brain waves), muscle relaxation monitoring, etc.), intradermal reaction

test (patch test, intradermal test, scratch test, tuberclin reaction, minimum erythema dose measurement, etc.), immunological examinations (viral tests, etc.) 7

（3）【処置】 静脈血採血，動脈血採血，動脈留置針挿入，痰等の吸引，胃管カテーテル，膀胱留 置カテーテル，う歯(虫歯)，歯周病，義歯の検査と治療，口腔ケア等の処置，マウスピース作成・装脱着，チューブやドレーン類の固定・脱着，創傷の処置，抜糸，抜 釘，小皮膚切開，縫合，ドレーン挿入・抜去，簡単なデブリードマン，涙管洗浄，ブ ジ挿入，鼻内処置，口内処置，睫毛脱毛，爪切り，点眼処置

[Treatment Measures] Venous blood collection, arterial blood collection, arterial indwelling needle insertion, phlegm aspiration, gastrointestinal catheter, bladder indwelling catheter, examination and treatment of dental caries , periodontal disease, prosthetic tooth, oral care treatment measures, preparation・ attachment / detachment of mouthpieces , fixation / detachment of tubes and drains, wound treatment, suture removal, nail removal, small skin incision, suturing, drain insertion / removal, simple debridement, lacrimal duct cleaning, bougienage, intranasal treatment measures, oral cavity treatment measures, eyelash hair removal, nail clipping, eye drops

（4）【投薬，投与】 通常の投薬，注射，末梢静脈内留置針挿入（点滴路の確保），持続皮下留置針挿入， 酸素投与等 上記の診療行為は，一定以上の経験を有する者によって行われますが，それでも時 に出血，痺れ，アナフィラキシー，その他予期せぬ合併症を伴うことがあり得ます。 このような場合は，合併症の治療は通常の保険診療として行われます。あらかじめご理解いただきますよう，お願いいたします。

[Medication dosing and administration] Ordinary medication dosing, injection, insertion of peripheral intravenous indwelling needle (securing a drip path), continuous subcutaneous indwelling needle insertion, oxygen administration, etc. Although the above medical procedures are performed by someone with a certain level of experience, occasionally there maybe bleeding, numbness, anaphylaxis, and other unexpected complications. In such cases, complications are treated as a regular health insurance. We appreciate your understanding in advance.

（5）【診療のために必要な画像等】 診察室，手術室，ＩＣＵ（集中治療室），ＨＣＵ，ＣＣＵ，南４病棟ＬＤ室，ＭＦ 室，放射線治療室，ＭＲＩ室，ＣＴ室，透視室では，治療上必要な場合は，患者さん の検査，診療行為，歩行等の様子をカメラ等による撮影，録音，記録，保管させてい ただいております。

[Imaging required for medical treatment] In the examination room, operating room, ICU (intensive care unit), HCU, CCU, south 4th ward LD room, MF room, radiotherapy room, MRI room, CT room, fluoroscopy room, if necessary for treatment, we use cameras to record and store pictures, record voice, record video of patient's medical examination and treatment action, walking behavior, etc.

４．学生等の実習について

本院は，診療を行う医療機関であると共に，教育研究機関として医療の「教育」と「研究」

を使命とし，次世代を担う医療人育成を行っております。その一環として，学生等の実習生を受け入れて，教員指導下に教育を行っており，学生等の実習生が診察等 に同席させていただくことがあります。患者さんには，ご理解とご協力をいただきます ようお願いいたします。

Regarding practical training for students, etc. This hospital is a medical institution that provides medical care, and functioning simultaneously as an educational and research institution, with the mission of medical "education" and "research", we are training medical personnel who will lead the next generation. As one component of this, we accept trainees such as students and provide education under the guidance of teachers, and trainees such as students may attend during medical examinations. We solicit the understanding and cooperation of patients.

５．診療に伴い発生する情報の利用について

現在，本院が提供している治療は，現時点でもっとも良いと科学的に評価されている治療法が中心になっています。医療は，研究を積み重ねることによって進歩を続けてきました。そのため，新しい治療法や診断法等を開発する研究には，患者さんから得られ る情報が不可欠です。本院でも，診療を行うとともに医学の発展に寄与するような臨床 研究や基礎研究を行っており，その際には，診療に伴って発生する情報を利用すること が必要な場合があります。 患者さんには，ご理解とご協力をいただきますようお願いいたします。

Regarding the usage of information generated during the medical treatment Currently, the treatments provided in this hospital are mainly those that are scientifically evaluated as the best available at the moment. Medical care has continued to advance through the accumulation of research. Therefore, information obtained from patients is indispensable for research to develop new treatment and diagnostic methods. At this hospital, besides being engaged in medical treatment, we also carry out clinical research and basic research that contribute to the development of medicine. As such, it may be necessary to use the information generated during the medical treatment. We solicit the understanding and cooperation of patients.

（１）【診療に伴い発生する情報とは】 本院で診療を受けると，診療録（カルテ），Ｘ線写真，患部の画像等のデータ，血液 検査，病理検査，生理機能検査結果（心電図，各種超音波検査，聴力検査，平衡機能 検査，呼吸機能検査等）の診療情報が集められます。 また，血液や尿等の検査試料，診断のための生検（胃内視鏡検査等の際に組織の一 部を採取すること）した試料，手術で切除した組織等の試料が集められます。 これらの集められた診療情報及び試料は，診療に必要なものとして，保管されてい ますが，その後，診療上不必要となった場合でも，医学研究・教育のための大切な情報となります。 本院では，これらの情報を病気の発生，進行，再発，予後等との関わりや，病気に 伴う症状，治療の効果，副作用等について研究する目的で利用することがあります。

[What is the information generated by medical treatment] When patients receive medical treatment at this hospital, medical information such as medical records, X-rays, data such as images of the affected areas, blood tests, pathological tests, physiological function tests

results (electrocardiogram, various ultrasonic tests, hearing tests, balance function tests, respiratory function tests), are collected. In addition, test samples such as blood and urine, biopsy samples for diagnosis (collecting a part of tissue at the time of gastroscopy, etc.), samples of tissue excised by surgery, etc. are collected. These collected medical information and samples are stored as necessity for medical treatment; but even if they become unnecessary for medical treatment subsequently, they will be important information for medical research and education. At our hospital, we may use this information for the purpose of studying the relationship between the onset, progression, recurrence, prognosis of the disease, the symptoms associated with the disease, the effects of treatment, side effects, etc.

（２）【情報の提供】 本院では，すべての患者さんにこれらの情報のご提供をお願いしています。この情報提供は，自由意思によります。原則として，不同意の意思表示がない場合は，同意 をいただいたものとみなして医学教育・研究に利用させていただきます。 [Provision of information] We request all patients to provide this information. This information is provided at free will. As a general rule, if there is no manifestation of disagreement, we will consider it as a consent and use it for medical education and research.

（３）【秘密の保持】 情報の提供によって，患者さんに直接の危険性が及ぶことはありません。しかし，万が一個人情報等が漏れてしまった場合は，患者さんが不利益を被る可能性がありま す。このため，本院では，考え得る危険性を避けるため，厳重な機密保持の方策を採 っています。情報は匿名化して扱いますので，研究責任者及び関係者以外は，患者さ んの氏名等と結びつけることはできません。また，研究によって得られた成果を学会 や科学専門誌等に発表する場合にも，個人が特定されることはありません。

[Maintaining confidentiality] Providing information does not pose a direct risk to the patient. However, in the unlikely event that personal information is leaked, the patient may suffer disadvantages. For this reason, our hospital has taken strict confidentiality measures to avoid potential dangers. Since the information is handled anonymously, it cannot be linked to the patient's name, etc., except for the principal investigator and related parties. In addition, no individual is identified when the results obtained from the research are published in academic societies or scientific journals.

６．包括同意について

ご不明な点がある場合又は意思表示をされる場合につきましては，医療安全相談室（外来棟１階中央窓口）にご相談ください。それぞれの担 当部署等をご案内いたします。

In case you have any questions regarding the comprehensive consent or if you have a manifestation of intention, please feel free to contact the Medical Safety Counseling Room (Central Reception on the 1st floor of the Outpatient Building). We will guide you to each department in charge.

Certificate of Consent

I have read the foregoing information, or it has been read to me. I have had the opportunity to ask questions about it and any questions that I have asked have been answered to my satisfaction.

I consent voluntarily to participate as a participant in this research.

Print Name of Participant__________________

Signature of Participant ___________________

Date ____________________________

Day/month/year

If illiterate

A literate witness must sign (if possible, this person should be selected by the participant and should have no connection to the research team).

I have witnessed the accurate reading of the consent form to the potential participant, and the individual has had the opportunity to ask questions. I confirm that the individual has given consent freely.

Print name of witness_____________________

Signature of witness ______________________

Date _________________________

Day/month/year

Statement by the researcher/person taking consent

I have accurately read out the information sheet to the potential participant, and to the best of my ability made sure that the participant understands that the following will be done:

I confirm that the participant was given an opportunity to ask questions about the study, and all the questions asked by the participant have been answered correctly and to the best of my ability. I confirm that the individual has not been coerced into giving consent, and the consent has been given freely and voluntarily.

Print Name of Researcher/person taking the consent__________________________

Signature of Researcher /person taking the consent__________________________

Date ____________________________

Day/month/year
