# Supplementary material for: Cardiopulmonary Events of the Elderly (≥75 Years) during Clazosentan Therapy after Subarachnoid Hemorrhage: A Retrospective Study from a Tertiary Stroke Center in Japan
Source: Medicina (Kaunas). 2024 Jan 21;60(1):185. doi: 10.3390/medicina60010185 (PMC10819954; doi:10.3390/medicina60010185)
Supplement: Supplementary file 1 [file medicina-60-00185-s001.zip › Table S1_medicina-2816537R1.pdf]

Table S1. Baseline characteristics and univariate analysis for clazosentan therapy between completed and discontinued groups among 33 consecutive SAH patients.

	Total (n=33)	Completed (n=26)	Discontinued (n=7)	*P*
Age (y)	63.5 (14.0)	60.8 (12.9)	67.0 (15.3)	0.29
Elderly (≥75 y), n	6 (18)	3 (12)	3 (43)	
Younger (<75 y), n	27 (82)	23 (88)	4 (57)	
Women	26 (79)	20 (77)	6 (86)	0.53
Body weight (kg)	59.4 (11.6)	60.0 (12.0)	58.8 (11.5)	0.13
WFNS grade	2.2 (1.5)	2.4 (1.6)	2.0 (1.5)	0.54
Location of aneurysms				
Anterior circulation	31 (94)	24 (92)	7 (100)	
Posterior circulation	2 (6)	2 (8)	0 (0)	
Treatment				
Endovascular coiling	9 (33)	8 (31)	1 (14)	
Surgical clipping	22 (67)	16 (69)	6 (86)	
Duration of drug therapy	8.7 (2.2)	13.1 (1.7)	4.7 (2.4)	**<0.001**
Minimal urine volume				
24-h volume (mL/d)	1,730 (592)	2,171 (747)	1,285 (354)	**0.005**
Volume per hour (mL/kg/h)	1.17 (0.68)	1.37 (0.85)	0.95 (0.31)	**0.02**
Day-to-day variance (mL/kg/h)	-0.75 (0.53)	-0.51 (0.55)	-0.96 (0.49)	**0.04**
Daily fluid balance at minimal urine volume(mL/d)	1,995 (826)	1,965 (796)	2,068 (878)	0.77
Weight gain (kg)	2.7 (1.6)	2.2 (1.4)	3.2 (1.8)	0.09
Vasospasm	9 (27)	6 (23)	3 (43)	0.28
DCI	5 (15)	3 (12)	2 (29)	0.28
90-d mRS 0–2	25 (76)	20 (77)	5 (71)	0.61

Data are shown as mean (standard deviation) or number (percentage). Statistically significant results at $P < 0.05$ are shown in bold.
DCI, delayed cerebral ischemia; eGFR, estimated glomerular filtration rate; mRS, modified Rankin scale; SAH, subarachnoid hemorrhage; WFNS, World Federation of Neurosurgical Societies.
